# Property of Phytosterols and Development of Its Containing Mayonnaise-Type Dressing

**DOI:** 10.3390/foods11081141

**Published:** 2022-04-14

**Authors:** Ryosuke Matsuoka

**Affiliations:** R&D Division, Kewpie Corporation, Tokyo 182-0002, Japan; ryosuke_matsuoka@kewpie.co.jp; Tel.: +81-3-5384-7759

**Keywords:** phytosterol esters, free phytosterols, mayonnaise-type dressing, cholesterol

## Abstract

Phytosterols are functional ingredients with known efficacy and safety. Phytosterols are found as free sterols or as their esters with fatty acids. Although phytosterol esters are soluble in oil and have been used in many commercial foods, it has been difficult to similarly use free phytosterols since they are insoluble in water and practically insoluble in oil. We have developed mayonnaise-type dressing materials using free phytosterols since people who are conscious about cholesterol intake are likely to be conscious about oil. In this review article, we summarized pieces of evidence for the development of phytosterol-containing mayonnaise-type dressing materials.

## 1. Introduction

Mayonnaise is made mainly of edible vegetable oil, egg yolk, and acetic acid. In Japan, raw materials for mayonnaise production are strictly specified by the Japan Agricultural Standard (JAS) [1]. Dressing materials made with raw materials not listed in the JAS (such as semisolid dressings and creamy salad dressings) are commonly referred to as “mayonnaise-type” dressing materials.

Since raw materials for mayonnaise production include egg yolk, people with hypercholesterolemia tend to refrain from using mayonnaise in their diets [2]. Although the edible vegetable oils in mayonnaise have been reported to reduce serum cholesterol levels [2,3], cholesterol-containing foods with functional ingredients effective for lowering the serum cholesterol levels help diversify food options for people with hypercholesterolemia.

Edible vegetable oil is a raw ingredient used in mayonnaise production. It accounts for ~70% of mayonnaise, making mayonnaise a relatively high-energy food [2]. Reducing energy intake is an important element of dietary instructions for people with hypercholesterolemia. Therefore, we planned to develop low-energy mayonnaise-type dressing materials containing functional ingredients to lower serum cholesterol levels.

Functional ingredients known to reduce serum cholesterol levels include dietary fiber, chitosan, low-molecular-weight alginic acid, catechin, soybean proteins, egg white proteins, and phytosterols [4,5,6,7,8,9,10,11,12,13].

Given that the adequate daily intake of mayonnaise is low (15 g), phytosterols were selected as functional ingredients since they are effective at lower doses than other serum cholesterol-lowering functional ingredients.

Phytosterols are found as free sterols or their esters with fatty acids [14,15]. Regardless of the form in which a phytosterol is ingested, the free form is responsible for the serum cholesterol-lowering effect [14,15]. Therefore, we adopted free sterols that are smaller in molecular weight and require smaller amounts to exert the effect.

Phytosterols are attractive, functional ingredients for preventing arteriosclerotic diseases since they reduce serum total cholesterol and LDL-cholesterol levels. In this review article, we summarize findings relevant to the development of phytosterol-containing mayonnaise-type dressing materials.

## 2. Phytosterols

**Figure 1:**
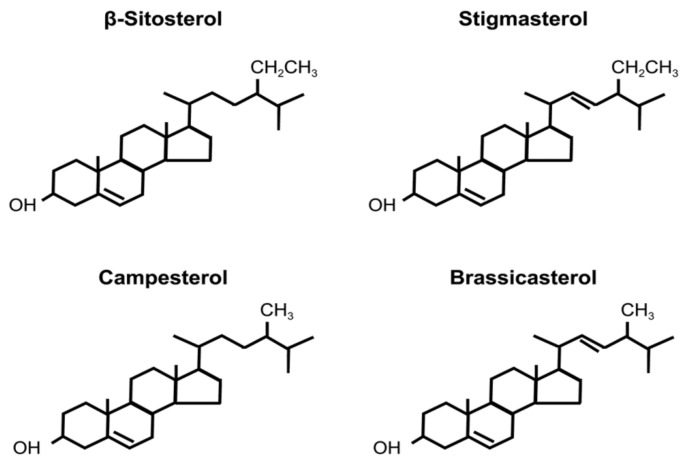
Structure of phytosterols.

Phytosterols are structurally similar to cholesterol and constitute plant cell membranes [16]. Various phytosterols are found in nature, especially in seeds, major examples of which include β-sitosterol, campesterol, stigmasterol, and brassicasterol [17]. Structures of these phytosterols are shown in Figure 1.

## 3. Consumption of Phytosterols

Since phytosterols constitute plant cell membranes and are thus contained in most plant foods, humans routinely ingest phytosterols as constituents of plant foods, such as vegetables, grains, fruits, and vegetable oils. Phytosterols are particularly abundant in vegetable oils, cacao beans, peanuts, and broccoli [18].

Daily intake amounts of phytosterols in Japanese have been reported to be 400 mg by Nakashima et al. through a survey of university cafeterias [19] and 0.373 g by Hirai et al. [20]. The daily intake of phytosterols in children aged 6–12 years was 137 ± 65 mg according to a survey by Ishinaga et al. [21].

The following values have been described in reports from other countries. Nair et al. surveyed the suburbs of Los Angeles and reported that daily intake amounts of phytosterols in the general public and lacto-ovo vegetarian populations were 0.078 g and 0.344 g, respectively [22]. Morton et al. have reported that the phytosterol intake in British people was 0.158 g [23]. The mean daily intake of phytosterols in Dutch people was 0.289 g [24].

Furthermore, as described previously, humans worldwide have a history of daily exposure to phytosterols as constituents of plant foods since ancient times. They are thus considered to have a sufficient eating experience.

## 4. Safety of Phytosterols

Phytosterol safety studies focusing on acute toxicity, subacute toxicity, chronic toxicity, mutagenicity, and reproductive toxicity have shown no toxic aberrance [25,26,27,28,29,30,31]. No serious adverse reactions were found in healthy individuals, postmenopausal women, and infants with familial hypercholesterolemia after phytosterols ingestion [31,32,33,34,35].

Phytosterols reduce blood levels of some fat-soluble vitamins within the normal ranges and thus have no major effects on serum levels of fat-soluble vitamins [34,35,36,37]. In addition, phytosterols had no effects on levels of fat-soluble vitamins in the blood [14,32,33,38,39].

In healthy humans, phytosterol ingestion increases serum β-sitosterol levels only slightly. The concentration of β-sitosterol in human blood has been reported to be less than 1 mg/dL [40], which did not change after phytosterol-containing mayonnaise-type dressing materials were ingested [14,33]. The concentration was markedly lower (~1/100) than the serum β-sitosterol level in patients with β-sitosterolemia, which causes pathological symptoms [41]. Phytosterols are highly safe since they are hardly absorbed by the body [42,43,44]. Even if phytosterols are absorbed by the body from the small intestine and migrate into the blood, phytosterols are considered safe in normal humans since they are discharged from the body [45]. These data indicate that phytosterols are highly safe materials.

Moreover, phytosterols are recognized as safe. They are listed as existing food additives in Japan, and fatty acid esters of phytosterols are certified as generally recognized as safe substances in the United States.

## 5. Serum Cholesterol-Lowering Effect of Phytosterols

After SD rats ingested β-sitosterol, a phytosterol, along with cholesterol, the total serum cholesterol and liver cholesterol levels decreased [46]. In male CD-1 mice fed with a diet containing 1% β-sitosterol for 15 days, the liver cholesterol level decreased significantly compared with controls. No significant differences in the serum total cholesterol level were observed. In addition, although the fecal sterol excretion increased, bile acid excretion significantly decreased [47].

Golden Syrian hamsters fed with a diet containing 0.25% cholesterol and 0.01%, 0.2%, or 1% phytosterols for 45 days, and those receiving ≥0.2% phytosterols showed significant decreases in the serum and liver cholesterol levels [48].

In rats that underwent thoracic duct cannulation, administration of β-sitosterol along with cholesterol significantly reduced the amount of cholesterol recovered in the lymph [49]. When phytosterols (a mixture of β-sitosterol, campesterol, stigmasterol, and brassicasterol) were administered with cholesterol to Wistar rats, serum and liver cholesterol levels decreased, and the fecal excretion of neutral sterols increased [50].

Ooyama et al. investigated the effects of phytosterols on postprandial serum lipid levels in healthy Japanese individuals who received chicken eggs and phytosterol-containing edible oil. The results showed that phytosterol-containing edible oil suppressed the increase in postprandial RLP-C levels [51].

The data described in these reports have revealed that inhibition of cholesterol absorption from the small intestine underlies the serum total cholesterol-lowering effect of phytosterols.

## 6. Mechanisms Underlying Cholesterol Absorption Inhibition by Phytosterols

**Figure 2:**
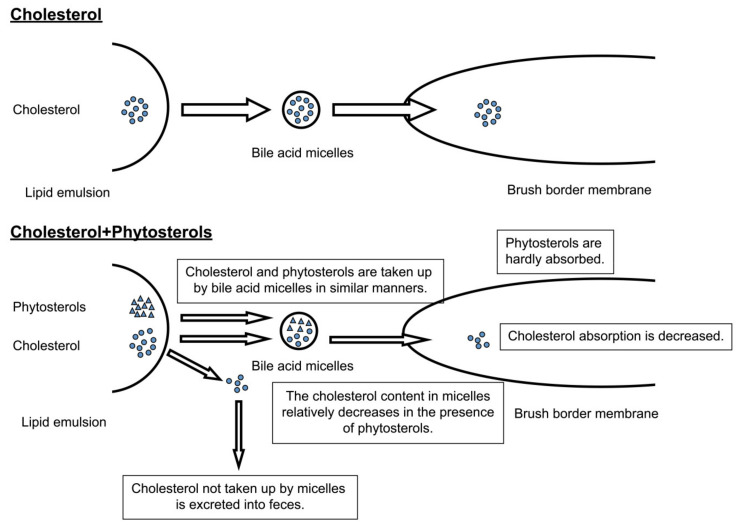
Mechanism for inhibition of cholesterol absorption of phytosterols [16].

Complex mechanisms underpin digestion/absorption of cholesterol. Dietary cholesterol is found as free cholesterol or a cholesterol ester with a fatty acid. A cholesterol ester is hydrolyzed to free cholesterol and fatty acid in the small intestine lumen by the action of cholesterol esterase secreted by the pancreas. Hydrolyzed cholesterol forms micelles with bile acids, phospholipids, and fatty acids. The micelles enter a water layer that does not mix with the small intestine contents and is located adjacent to the microvillus membrane (unstirred water layer). In this layer, micelles release cholesterol molecules as monomers, which are taken up via simple diffusion according to a cholesterol concentration gradient across the microvillus membrane. Cholesterol molecules that are taken up by the cells are reconverted to esters with fatty acids by acyl-CoA cholesterol acyltransferase (ACAT). The resulting cholesterol esters are incorporated into chylomicrons and released into the lymph [16] (Figure 2).

Phytosterols inhibit cholesterol absorption through a series of steps, as summarized below. Possible steps responsible for the inhibition are as follows [16]. Figure 1 shows the mechanism by which phytosterols inhibit the absorption of cholesterol.

1. Phytosterols compete against cholesterol in micelle formation, resulting in inhibition.
2. Phytosterols bind to and inhibit the cholesterol receptor essential for the cholesterol uptake across the microvillus membrane.
3. Phytosterols inhibit the intracellular esterification of cholesterol.

First, β-sitosterol, a phytosterol, has been reported to form micelles just as cholesterol does. Since only a limited amount of sterols is dissolved in micelles, the coexistence of cholesterol and β-sitosterol in the lumen of the small intestine results in a relative decrease in the cholesterol solubility in the micelle [42,49,52]. Furthermore, β-sitosterol is hardly absorbed from the small intestine [42,43] and is retained in micelles and emulsions. As a consequence, cholesterol solubility in micelles is reduced. This contributes to the decreased cholesterol absorption since cholesterol solubilization in micelles is essential for its absorption. Moreover, in rats fed with a diet containing cholesterol alone and those fed with a diet containing cholesterol and β-sitosterol, β-sitosterol decreased the cholesterol solubility in micelles, the cellular cholesterol uptake via the microvillus membrane in the small intestine [16,52], and the cholesterol recovery in the lymph by practically the same percentages [16,49]. These results demonstrate that phytosterols inhibit cholesterol absorption during cholesterol incorporation into micelles.

Second, β-sitosterol does not specifically bind to the microvillus membrane and does not competitively inhibit cholesterol absorption [16,49]. Third, β-sitosterol has no inhibitory effect on cholesterol esterification in mucosal cells of the small intestine [16,49].

Therefore, phytosterols inhibit cholesterol absorption by reducing the cholesterol solubility in micelles, lowering the serum cholesterol level.

In addition to inhibiting cholesterol absorption by phytosterols, recent findings have indicated that small amounts of phytosterols are absorbed and reach the liver. Plant sterols that reach the liver promote cholesterol’s catabolism into bile acids and excretion into bile by activating the Liver X receptor, α-CYP7A1 pathway [53]. Phytosterols reduced hepatic cholesterol level [15]. These effects reduce serum LDL cholesterol levels by reducing the hepatic cholesterol pool and the excretion of cholesterol into the blood by VLDL from the liver [54].

## 7. Minimum Effective Dose of Phytosterols

Pelletier et al. conducted a crossover study on 12 healthy individuals [53]. The participants ingested a butter preparation containing 740-mg/day phytosterols and a butter preparation containing no phytosterols every day for 4 weeks. When phytosterols were ingested, the total serum cholesterol and LDL-cholesterol levels were significantly lower than those when phytosterols were not ingested [55].

Sierksma et al. and Hendriks et al. tested the ingestion of 0.8- and 0.83-g/day phytosterols, respectively, and found significant decreases in the serum total cholesterol level [37,56]. Meanwhile, Miettnen et al. reported no significant difference between 0.63 and 0.65-g/day β-sitosterol and control mayonnaise [57].

**Figure 3:**
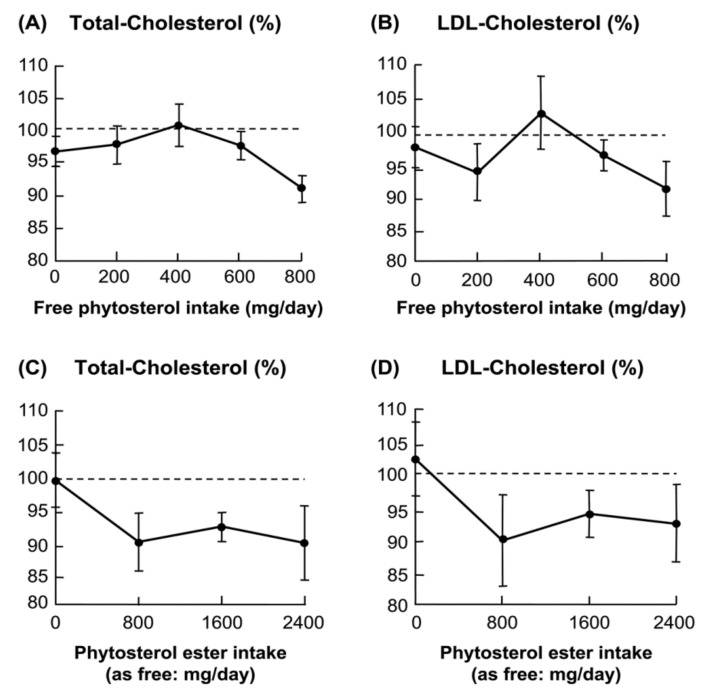
Minimal effective dose of phytosterol-containing mayonnaise-type dressing material in subjects. The subjects fed mayonnaise containing 0–2400 mg of plant sterol for four weeks. Mean ± SE. of 7–9 subjects [14].

Matsuoka et al. studied the minimum effective dose of phytosterols on serum cholesterol levels in Japanese participants. The results showed that the minimum effective daily dose of phytosterols for the serum cholesterol-lowering effect was likely to be 800 mg (Figure 3) [14]. In an extensive literature survey including nearly 70 articles on the phytosterol intake and serum cholesterol levels, Saito et al. concluded that the adequate intake of phytosterols was ≥800 mg [58].

These studies have shown that the adequate intake of phytosterols for serum cholesterol-lowering effect is ≥800 mg per day.

## 8. Free Phytosterol-Containing Mayonnaise-Type Dressing Materials

**Figure 4:**
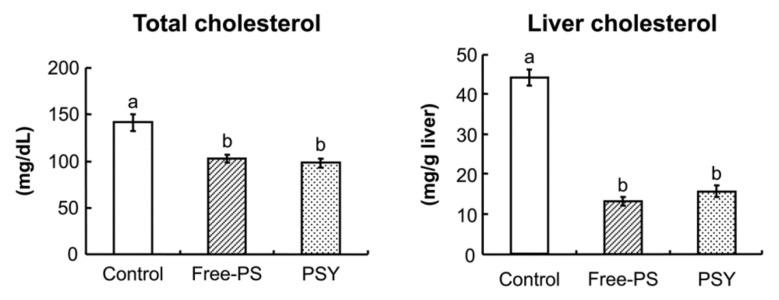
Cholesterol-lowering action of phytosterol egg yolk-lipoprotein complex in rat. Mean ± SE of six rats. Different letters shown significant difference (*p* < 0.05). PS, phytosterols; PSY, phytosterol egg yolk-lipoprotein complex [15].

People who are conscious about cholesterol in mayonnaise are often conscious about the oil present in mayonnaise. The phytosterol-containing mayonnaise-type dressing materials reported previously contained the same amount of oil as usual mayonnaise. Recently, low-energy mayonnaise-type dressing materials supplemented with fatty acid-free free phytosterols have been studied. First, to supplement a low-energy mayonnaise-type dressing material with phytosterols, phytosterols are required to form a complex with egg yolk. This phytosterol–egg yolk lipoprotein complex has been reported to reduce the serum and liver cholesterol level (Figure 4) [15]. Second, mayonnaise ingredients have been confirmed to have no negative effects on the cholesterol-lowering effect of phytosterols [50].

Table 1 shows the analytical composition of phytosterol-containing mayonnaise-type dressing

## 9. Safety of Phytosterol-Containing Mayonnaise-Type Dressing Materials

Twenty-three healthy men ingested 15 g of a phytosterol-containing mayonnaise-type dressing material (phytosterols 800 mg) daily for 12 weeks. Compared with the pre-ingestion values, the leukocyte count and LDH (lactate dehydrogenase) increased significantly statistically. The platelet count and HbA1C level decreased significantly statistically. However, all observed changes were within the respective normal ranges. General laboratory test values did not change significantly while the subjects ingested the phytosterol-containing mayonnaise-type dressing material. No significant changes were observed in serum vitamin A, 25(OH)-vitamin D, and vitamin K1 levels. The serum α-tocopherol level decreased significantly compared with the pre-ingestion level but remained normal in all subjects. Moreover, the serum β-sitosterol level increased significantly but was less than 1 mg/dL in all subjects [33].

Ten healthy men and five healthy women ingested 45-g/day phytosterol-containing mayonnaise-type dressing material (phytosterol 2400 mg), equivalent to three times the adequate daily intake, every day for four weeks, and their blood samples were collected before and after the ingestion period. The blood test results showed no significant changes in serum vitamin A and vitamin E levels. The serum β-sitosterol level showed a statistically significant increase but remained normal for all subjects. Phytosterols showed no effects on hepatic and renal function indicators and hematological test results. Furthermore, no changes in weight, body fat percentage, and blood pressure were observed [14].

High serum β-sitosterol levels are a risk factor for coronary artery diseases. The elevated serum β-sitosterol levels were seen in some subjects in the phytosterol-containing mayonnaise-type dressing material ingestion study. Meanwhile, there are some conflicting reports on this matter, and details remain to be clarified. Therefore, we will continue to monitor the relationship between the serum phytosterol level and coronary artery diseases and conduct studies if necessary.

In summary, these findings indicate that phytosterol-containing mayonnaise-type dressing materials are a highly safe food.

## 10. Efficacy of Phytosterol-Containing Mayonnaise-Type Dressing Materials

In Japanese men with mild hypercholesterolemia, effects of daily ingestion of a 15-g low-energy mayonnaise-type dressing material supplemented with 800-mg phytosterols for 12 weeks were compared with those of a mayonnaise-type dressing material containing no phytosterols. The serum total and LDL-cholesterol levels in the phytosterol-containing mayonnaise-type dressing material group were significantly lower than those in the control mayonnaise-type dressing material group. Moreover, the serum total and LDL-cholesterol levels after ingestion of the phytosterol-containing mayonnaise-type dressing material were decreased by 4.3% and 9.2%, respectively, compared with the pre-ingestion levels [33].

Effects of the phytosterol-containing mayonnaise-type dressing material (phytosterols 885 mg) were compared with those of a control mayonnaise-type dressing material supplemented with no phytosterols in 61 Japanese men who ingested these materials for 12 weeks. In subjects whose serum total cholesterol and LDL-cholesterol levels were ≥200 and ≥120 mg/dL, respectively. The serum total cholesterol levels at weeks 4, 8, and 12 and the serum LDL-cholesterol and ApoB levels at weeks 8 and 12 in the phytosterol-containing mayonnaise-type dressing material group were significantly lower than the pre-ingestion levels or values at the corresponding time points in the control mayonnaise-type dressing material group. Moreover, the serum total and LDL-cholesterol levels after ingestion of the phytosterol-containing mayonnaise-type dressing material were decreased by ~5% and 7–11%, respectively, compared with the pre-ingestion levels [59].

For the serum lipid measurements in subjects whose serum total cholesterol and LDL-cholesterol levels were ≥200 and ≥120 mg/dL, respectively, analysis of covariance was conducted using the initial value of each parameter as a covariate. The result revealed that the serum total cholesterol levels at weeks 4, 8, and 12 and the serum LDL-cholesterol and ApoB levels at weeks 8 and 12 in the phytosterol-containing mayonnaise-type dressing material group were significantly lower than the pre-ingestion levels or values at the corresponding time points in the control mayonnaise-type dressing material group (Table 2) [59].

Effects of the phytosterol-containing mayonnaise-type dressing material (phytosterols 885 mg) were compared with those of a control mayonnaise-type dressing material containing no phytosterols in 60 Japanese men and women who ingested these materials for 12 weeks. The total serum cholesterol, LDL-cholesterol, and ApoB levels at 4 and more weeks of ingestion of the phytosterol-containing mayonnaise-type dressing material were significantly lower than the values after ingestion of the control mayonnaise-type dressing material [60].

The decreases in serum total and LDL-cholesterol levels observed after daily ingestion of 15-g mayonnaise-type dressing material containing 885-mg phytosterols described above have raised hopes of preventing coronary artery disorders with long-term ingestion of the phytosterol-containing mayonnaise-type dressing material.

## 11. Discussion

To summarize the results described in this review, free phytosterols, even as components of low-energy mayonnaise-type dressing materials, reduce the total serum cholesterol and LDL-cholesterol levels in people with elevated serum cholesterol levels.

Table 3 summarizes the evidence of phytosterols containing mayonnaise-type dressings (tested in groups of at least 10 people).

Ishizaki et al. have reported that phytosterol esters blended in regular mayonnaise (equivalent to 884-mg free phytosterols/15 g) also reduced total serum cholesterol and LDL-cholesterol levels [61]. Sato et al. have reported similar results with a low-energy mayonnaise-type dressing material containing phytosterol esters [62]. In addition, phytosterols at 400 mg per day combined with diacylglycerol and a mayonnaise-type dressing material containing these ingredients reduce total serum total cholesterol and LDL-cholesterol levels [63,64].

These results indicate that the effects of phytosterols to reduce total serum cholesterol and LDL-cholesterol levels are retained by those blended in mayonnaise or a low-energy mayonnaise-type dressing material. Furthermore, mayonnaise ingredients have been confirmed not to interfere with the effects of phytosterols (albeit in animal experiments) [50].

**Table 3:** Effects of plant sterol containing mayonnaise-type dressing on serum total and LDL-cholesterol levels in human.

Study	Subjects	Test Mayonnaise	Design Period	Main Outcome (Change% vs. before Intake)
Ishizaki, T. (2003) [61]	Normo and Mildly hypercholesterolemia (PSE: *n* = 26, placebo: *n* = 29)	Mayonnaise1-type dressing (PSE: 884 mg/15 g as free PS)	RCT 12 weeks	TC: −4.6%, LDL-C: −5.6%
Saito, S. (2006) [63]	Normo and Mildly hypercholesterolemia (*n* = 16–17)	Diacylglycerol based mayonnaise-type drsssing (Free PS: 0–500 mg/15 g)	RCT 4 weeks	300 mg: TC: −6.0%, LDL-C: −12.1% 400 mg: TC: −7.1%, LDL-C: −11.0% 500 mg: TC: −8.7%, LDL-C: −14.4%
Meguro, S. (2001) [64]	Normo and Mildly hypercholesterolemia (*n* = 12)	Diacylglycerol or triacylglycerol based mayonnaise-type dressing (Free PS: 500 mg/15 g)	Crossover 4 weeks	Triacylglycerol oil base TC, LDL-C: No change Diacylglycerol oil base TC: −4.6%, LDL-C: −8.1%
Matsuoka, R. (2004) [33]	Normo and Mildly hypercholesterolemia (Free-PS: *n* = 23, placebo: *n* = 23)	Low-energy Mayonnaise-type dressing (Free PS: 823 mg/15 g)	RCT 12 weeks	TC: −4.3%, LDL-C: −9.2%
Matsuoka, R. (2005) [59]	Normo and Mildly hypercholesterolemia (Free-PS: *n* = 30, placebo: *n* = 31)	Low-energy Mayonnaise-type dressing (Free PS: 885 mg/15 g)	RCT 12 weeks	Total subjcts: TC: −3.4%, LDL-C: −4.7% Mildly hypercholesterolemia: TC: −5.0%, LDL-C: −7.8%
Takeda, S. (2008) [60]	Normo and Mildly hypercholesterolemia (Free PS: *n* = 27, placebo: *n* = 27)	Low-energy Mayonnaise-type dressing (Free PS: 885 mg/15 g)	RCT 12 weeks	TC: −5.1%, LDL-C: −5.2%
Sato, H. (2010) [62]	Normo and Mildly hypercholesterolemia (PSE: *n* = 45, placebo: *n* = 35)	Low-energy Mayonnaise-type dressing (PSE: 884 mg/15 g as free PS)	RCT 12 weeks	TC: −5.7%, LDL-C: −6.5%

TC, total cholesterol; LDL-C, LDL-cholesterol; RCT, randomized controlled study; PS, plant sterols; PSE, plant sterol esters.

Aside from mayonnaise, phytosterols are also used in various other foods. In particular, margarine, cooking oil, yogurt, soy milk, low-fat milk, juice, milk tea, and dressings supplemented with phytosterols reduce the total serum cholesterol and LDL-cholesterol levels [36,39,48,51,55,65,66,67,68,69,70,71,72,73,74,75]. Phytosterols can be blended in foods in a limited variety of forms since they are insoluble in water and practically insoluble in oil. However, the data suggest that the effects of plant sterols are not affected substantially by foods that can be supplemented with phytosterols.

In addition to the eating experience of phytosterols, the distribution of many phytosterol-containing foods for more than 20 years had no reports raising safety concerns supports the safety of phytosterols. Furthermore, phytosterols can be used reliably to reduce total serum cholesterol and LDL-cholesterol levels since their mechanisms of action and metabolism have been demonstrated [16,45,52].

Elevation of the serum LDL-cholesterol level is a risk factor for atherosclerotic diseases, such as myocardial infarction and cerebral infarction. Since cerebral infarction can cause paralysis and shorten healthy life expectancy, counteracting the elevation of the serum LDL-cholesterol level is also important for preventing these diseases.

Although this review article concerns mayonnaise-type dressing materials supplemented with phytosterols, mayonnaise is a dressing for vegetable salads in the first place. Vegetables contain many substances that reduce serum cholesterol levels such as dietary fiber (as do phytosterols) [4,5]. Aside from dietary fiber, many other serum cholesterol-lowering materials have been reported, including chitosan, low-molecular-weight alginic acid, catechin, soybean proteins, and egg white proteins [6,7,8,9,10,11,12,13]. Thus, the combined use of phytosterols with these ingredients may be a useful dietary means for preventing arteriosclerotic diseases. In consistent, several recent studies have also reported the blood cholesterol-lowering effects of phytosterols [76,77,78,79,80,81,82]. In addition, the anti-inflammatory and anti-diabetic effects of phytosterols further contribute to the prevention of cardiovascular disease [83,84,85]. Processed foods, such as mayonnaise-type dressing, which contain phytosterols and are significant and popular, are described in this review. We expect to develop the research further on phytosterols and new products using phytosterols in the future.

## 12. Conclusions

Phytosterol-containing mayonnaise has been proven to be safe and effective in reducing total serum cholesterol and LDL-cholesterol levels in people with elevated serum cholesterol levels, thereby contributing to the prevention of arteriosclerotic diseases and living a healthier life.

## Figures and Tables

**Table 1:** Analytical composition of phytosterol-containing mayonnaise-type dressing *.

Energy	(kcal)	48
Protein	(g)	0.3
Fat ^#^	(g)	4.9
Carbohydrate	(g)	0.5
Sodium	(mg)	165
Cholesterol	(mg)	22
Plant sterol	(mg)	885

*: Values are per 15 g of mayonnaise. ^#^: include plant sterols.

**Table 2:** Effects of phytosterol-containing mayonnaise-type dressing material on the normo- and mildly hypercholesterolemia (serum total cholesterol ≥ 200 mg/dL, and LDL-cholesterol ≥ 120 mg/dL).

	Group	Week				Statistical	+2 Week
		0	4	8	12	Significance ^a^	(14 Week)
TC (mg/dL)	PS (−)	247 ± 6	250 ± 6	246 ± 5	259 ± 7	NS	243 ± 5 ^†^
	PS (+)	244 ± 7	232 ± 7 *^,#^	229 ± 5 *^,#^	230 ± 6 *^,#^	*p* = 0.009	223 ± 5 ^#^
LDL-C (mg/dL)	PS (−)	159 ± 5	157 ± 6	154 ± 5	164 ± 7	NS	151 ± 6 ^†^
	PS (+)	156 ± 5	145 ± 5 *	138 ± 4 *^,#^	145 ± 4 *^,#^	*p* < 0.001	139 ± 4
Apo B (mg/dL)	PS (−)	127 ± 4	121 ± 4	123 ± 4	127 ± 5	NS	119 ± 4 ^†^
	PS (+)	121 ± 4	111 ± 4 *	111 ± 3 *^,#^	111 ± 4 *^,#^	*p* < 0.001	107 ± 3

TC, total cholesterol; LDL-C, LDL-cholesterol; ApoB, apolipoprotein B; PS, plant sterol. ^a^ Statistical significance by ANCOVA vs. 0 Week at *p* < 0.05. NS, not significant. Mean ± SE. of PS (−) group, *n* = 24; PS (+) group, *n* = 26. ^#^ Statistical significance by ANCOVA vs. PS (−) group at *p* < 0.05. * Statistical significance by ANCOVA and Dunnett test vs. 0 week at *p* < 0.05. ^†^ Statistical significance by Paired *t*-test vs. 12 week at *p* < 0.05.

## Data Availability

No new data were created or analyzed in this study. Data sharing is not applicable to this article.

## References

[1] Kimura T., Tanaka T., Umezu T., Ariizumi M. (2011). New technological development for functional mayonnaise. Jpn. J. Food Eng..

[2] Matsuoka R., Masuda Y., Takamiya M., Kawamura M., Hirayama S., Hasegawa M., Tohgi N. (2001). Effect of mayonnaise on serum lipid concentrations in hyperlipidemic subjects. J. Jpn. Soc. Clin. Nutr..

[3] Karupaiah T., Chuah K.A., Chinna K., Matsuoka R., Masuda Y., Sundram K., Sugano M. (2016). Comparison of soybean oil- and palm olein-based mayonnaise on the plasma lipid and lipoprotein profiles in human subjects: A double-blind randomized controlled trial with cross-over design. Lipids Health Dis..

[4] Mia M.A., Siddiqui M.N., Haque M.S., Islam M.N., Rukunzzaman M., Deb K. (2002). Dietary fibre and coronary heart disease. Mymensingh Med. J..

[5] Bell L.P., Hectorn K.J., Reynolds H., Hunninghake D.B. (1990). Cholesterol-lowering effects of soluble-fiber cereals as part of a prudent diet for patients with mild to moderate hypercholesterolemia. Am. J. Clin. Nutr..

[6] Maezaki Y., Tsuji K., Nakagawa Y., Kawai Y., Akimoto M., Tsugita T., Takekawa W., Terada A., Hara H., Mitsuoka T. (1993). Hypocholesterolemic effect of chitosan in adult males. Biosci. Biotechnol. Biochem..

[7] Ikeguchi M., Kobayashi M., Ariura Y., Mitsui T., Takagaki K., Ishibashi Y., Tsuji K. (2003). The effect of AOJIRU drink powder of barley green grasses containing chitosan on the serum lipids and the safety. J. Nutr. Food.

[8] Nishizawa M., Iwata K., Yamagishi T., Tsuji K. (1997). Effect of depolymerized sodium alginate on the serum and liver cholesterol levels in cholesterol-fed rats. J. Home Econ. Jpn..

[9] Kajimoto O., Kajimoto Y., Yabune M., Nozawa A., Nagata K., Kakuda T. (2003). Tea catechins reduce serum cholesterol levels in mild and borderline hypercholesterolemia patients. J. Clin. Biochem. Nutr..

[10] Tsuzuki K., Asao H., Ban S., Ikematsu H., Shionoya K. (2003). Evaluation of the effect of prepared soymilk containing soy protein on serum lipids and the safety of its excessive intake. J. Nutr. Food.

[11] Sirtori C.R., Lovati M.R., Manzoni C., Monetti M., Pazzucconi F., Gatti E. (1995). Soy and cholesterol reduction: Clinical experience. J. Nutr..

[12] Matsuoka R., Usuda M., Masuda Y., Kunou M., Utsunomiya K. (2017). Lactic-fermented egg white reduced serum cholesterol concentrations in mildly hypercholesterolemic Japanese men: A double-blind, parallel-arm design. Lipids Health Dis..

[13] Matsuoka R., Kimura M., Muto A., Masuda Y., Sato M., Imaizumi K. (2008). Mechanism for the cholesterol-lowering action of egg white protein in rats. Biosci. Biotechnol. Biochem..

[14] Matsuoka R., Masuda Y., Takeuchi A., Marushima R., Onuki M. (2004). Minimal effective dose of plant sterol on serum cholesterol concentration in Japanese subjects and safety evaluation of plant sterol supplemented in mayonnaise. J. Oleo Sci..

[15] Matsuoka R., Muto A., Kimura M., Hoshina R., Wakamatsu T., Masuda Y. (2008). Cholesterol-lowering activity of plant sterol-egg yolk lipoprotein complex in rats. J. Oleo Sci..

[16] Ikeda I. (1991). Studies on mechanism of sterols absorption. Nippon Nougeikagaku Kaishi.

[17] Ohno Y., Hara I. (1981). New aspects on nutrition: (2) sterol. J. Jpn. Oil Chem. Soc..

[18] Hidaka K., Yoshida K., Izaki Y., Toda K. (1986). Vitamin E, cholesterol and fatty acids in foods determination of their content and Estimation of daily intake. J. Jpn. Soc. Nutr. Food Sci..

[19] Nakajima K., Ikeda I., Fuchigami K., Shiroishi Y., Sugano M., Yasue R., Matsumoto M. (1981). Nutritional composition of university set meal—Especially sterol and dietary fiber contents. Jap. J. Clin. Nutr..

[20] Hirai K., Shimazu C., Takezoe R., Ozeki Y. (1986). Cholesterol, phytosterol and polyunsaturated fatty acid levels in 1982 and 1957 Japanese diets. J. Nutr. Sci. Vitaminol..

[21] Ishinaga M., Mochizuki T., Ueda A., Ichi I., Nanatsue M., Oda M., Kishida N. (2001). Daily intakes of fatty acids, cholesterol and plant sterols by obese and non-obese school children. J. Jpn. Soc. Nutr. Food Sci..

[22] Nair P.P., Turjman N., Kessie G., Calkins B., Goodman G.T., Davidovitz H., Nimmagadda G. (1984). Diet, nutrition intake, and metabolism in populations at high and low risk for colon cancer. Dietary cholesterol, β-sitosterol, and stigmasterol. Am. J. Clin. Nutr..

[23] Morton G.M., Lee S.M., Buss D.H., Lawrance P. (1995). Intakes and major dietary sources of cholesterol and phytosterols in the British diet. J. Hum. Nutr. Diet..

[24] Normén A.L., Brants H.A.M., Voorrips L.E., Andersson H.A., van den Brandt P.A., Goldbohm R.A. (2001). Plant sterol intakes and colotectal cancer risk in the Netherlands cohort study on diet and cancer. Am. J. Clin. Nutr..

[25] Japan Pharmaceutical Information Center (1974). Soy unsaponifiable. Pharm. Mon..

[26] Hepburn P.A., Horner S.A., Smith M. (1999). Safety evaluation of phytosterol esters. Part 2. Subchronic 90-day oral toxicity study on phytosterol esters—A novel functional food. Food Chem. Toxicol..

[27] Shipley R.E., Pfeiffer R.R., Marsh M.M., Anderson R.C. (1958). Sitosterol feeding—Chronic animal and clinical toxicology and tissue analysis. Circ. Res..

[28] Wolfreys A.M., Hepburn P.A. (2002). Safety evaluation of phytosterol esters. Part 7. Assesment of mutagenic activity of phytosterols, phytosterol esters and the cholesterol derivative, 4-cholesten-3–one. Food Chem. Toxicol..

[29] Waalkens-Berendsen D.H., Wolterbeek A.P.M., Wijnands M.V.W., Richold M., Hepburn P.A. (1999). Safety evaluation of phytosterol esters. Part 3. Two-generation reproduction study in rats with phytosterol ester—A novel functional food. Food Chem. Toxicol..

[30] Baker V.A., Hepburn P.A., Kennedy S.J., Jones P.A., Lea L.J., Sumpter J.P., Ashby J. (1999). Safety evaluation of phytosterol esters. Part 1. Assessment of oestrogenicity using a combination of in vivo and in vitro assays. Food Chem. Toxicol..

[31] Ayesh R., Weststrate J.A., Drewitt P.N., Hepburn P.A. (1999). Safety evaluation of phytosterol esters. Part 5. Feacal short-chain fatty acid and microfloracontent, feacal Bacterial enzyme activity and serum female sex hormones in healthy normolipidemic volumteers consuming a controlled diet either with or without a phytosterol ester-enriched margarine. Food Chem. Toxicol..

[32] Davidson M.H., Maki K.C., Umporowicz D.M., Ingram K.A., Dicklin M.R., Schaefer E., Lane R.W., McNamara J.R., Ribaya-Mercado J.D., Perrone G. (2001). Safety and tolerability of esterified phytosterols administered in reduced-fat spread and salad dressing to healthy adult men and women. J. Am. Coll. Nutr..

[33] Matsuoka R., Masuda Y., Takeuchi A., Marushima R., Hasegawa M., Sakamoto A., Hirata H., Kajimoto O., Homma Y. (2004). A double-blind, placebo-controlled study on the effects of mayonnaise containing free plant sterol on serum cholesterol concentration; Safety evaluation for normocholesterolemic and mildly hypercholesterolemic Japanese subjects. J. Oleo Sci..

[34] Gylling H., Miettinen T.A. (1999). Cholesterol reduction by different plant sterol mixtures and with variable fat intake. Metabolism.

[35] Tammi A., Rönnemaa T., Gylling H., Rask-Nissilä L., Viikari J., Tuominen J., Pulkki K., Simell O. (2000). Plant stanol ester margarine lowers serum total and low-density lipoprotein cholesterol concentrations of healthy children: The STRIP project. Special Turku Coronary Risk Factors Intervention Project. J. Pediatr..

[36] Hallikainen M.A., Uusitupa M.I.J. (1999). Effects of 2 low-fat stanol ester-containing margarines on serum cholesterol concentrations as part of a low-fat diet in hypercholesteroleic subjects. Am. J. Clin. Nutr..

[37] Hendriks H.F.J., Weststrate J.A., van Vliet T., Meijer G.W. (1999). Spreads enriched with three different levels of vagetable oil sterols and degree of cholesterol lowering in normocholesterolemic and mildly hypercholesterolemic subjects. Eur. J. Clin. Nutr..

[38] Christiansen L.I., Lähteenmäki P.L.A., Mannelin M.R., Seppänen-Laakso T.E., Hiltunen R.V.K., Yliruusi J.K. (2001). Cholesterol-lowering effect of spreads enriched with microcrystallin plant sterols in hypercholesterolemic subjects. Eur. J. Nutr..

[39] Volpe R., Niittynen L., Korpela R., Sirtori C., Bucci A., Fraone N., Pazzucconi F. (2001). Effects of yoghurt enriched with plant sterols on serum lipids in patients with moderate hypercholesterolaemia. Br. J. Nutr..

[40] Hidaka H. (2001). Sitoterolemia. Nihon Rinsho.

[41] Salen G., Kwiterovich P.O., Shefer S., Tint G.S., Horak I., Shore V., Dayal B., Horak E. (1985). Increased plasma cholesterol and 5α-saturated plant sterol derivatives in subjects with sitosterolemia and xanthomatosis. J. Lipid Res..

[42] Ikeda I., Sugano M. (1998). Inhibition of cholesterol absorption by plant sterols for mass intervention. Curr. Opin. Lipidol..

[43] Heinemann T., Axtmann G., von Bergmann K. (1993). Comparison of intestinal absorption of cholesterol with different plant sterols in man. Eur. J. Clin. Investig..

[44] Ostlund R.E. (2002). Phytosterols in human nutrition. Annu. Rev. Nutr..

[45] Subbiah M.T.R., Kuksis A. (1973). Differences in metabolism of cholesterol and sitosterol following intravenous injection in rats. Biochim. Biophys. Acta.

[46] Sugano M., Morioka H., Ikeda I. (1977). A comparison of hypocholesterolemic activity of β-sitosterol and β-sitostanol in rats. J. Nutr..

[47] Uchida K., Mizuno H., Hirota K., Takeda K., Takeuchi N., Ishikawa Y. (1983). Effects of spinasterol and sitosterol on plasma and liver cholesterol levels and biliary and fecal sterol and bile acid excretions in mice. Jpn. J. Pharmacol..

[48] Ntanios F.Y., Jones P.J.H. (1998). Effects of variable dietary sitostanol concentrations on plasma lipid profile and phytosterol metabolism in hamsters. Biochim. Biophys. Acta.

[49] Ikeda I., Tanaka K., Sugano M., Vahouny G.V., Gallo L.L. (1988). Inhibition of cholesterol absorption in rats by plant sterols. J. Lipid Res..

[50] Ogino Y., Hayashi N., Kimura M., Takizawa K., Matsuoka R., Masuda Y., Hasegawa M. (2004). Effect of dietary plant sterols and/or mayonnaise supplementation on lipid metabolism in rats. J. Oleo Sci..

[51] Ooyama K., Seki S., Hidaka I., Yoshino H., Tsuji H., Taguchi N., Nakajima S., Kondo K. (2001). Effects of plant sterol-containing oil on postprandial serum lipids. Jpn. J. Nutr. Diet.

[52] Ikeda I., Sugano M. (1983). Some aspects of mechanism of inhibition of cholesterol absorption by β-sitosterol. Biochim. Biophys. Acta.

[53] Li X., Xin Y., Mo Y., Marozik P., He T., Guo H. (2022). The Bioavailability and Biological Activities of Phytosterols as Modulators of Cholesterol Metabolism. Molecules.

[54] Cedó L., Farràs M., Lee-Rueckert M., Escolà-Gil J.C. (2019). Molecular Insights into the Mechanisms Underlying the Cholesterol- Lowering Effects of Phytosterols. Curr. Med. Chem..

[55] Pelletier X., Belbraouet S., Mirabel D., Mordret F., Perrin J.L., Pages X., Debry G. (1995). A diet moderatery enriched in phytosterols lowers plasma cholesterol concentrations in normocholesterolemic humans. Ann. Nutr. Metab..

[56] Sierksma A., Weststrate J.A., Meijer G.W. (1999). Spreads enriched with plant sterols, either esterified 4,4-dimethylsterols of free 4-desmosterols, and plasma total- and LDL-cholesterol concentrations. Br. J. Nutr..

[57] Vanhanen H.T., Miettinen T.A. (1992). Effects of unsaturated and saturated dietary plant sterols on their serum contents. Clin. Chim. Acta.

[58] Saito S., Ikeda I., Sugano M. (2002). Effect of plant sterols and stanols on blood cholesterol level: Clinical evidence of minimum effective dose. J. Jpn. Soc. Nutr. Food Sci..

[59] Matsuoka R., Marushima R., Kurokawa M., Masuda Y., Hasegawa M., Takahama M., Onuki M., Sugano M. (2005). Effect of mayonnaise containing plant sterol in Japanese male subjects—Safety evaluation of long-term intake and clinical benefit for serum lipid concentrations. J. Metab. Clin. Nutr..

[60] Takeda S., Masuda Y., Kurokawa M., Matsuoka R., Marushima R., Hasegawa M., Homma Y. (2007). Effects of mayonnaise containing plant sterol on blood cholesterol in borderline or mildly hypercholesterolemic Japanese subjects. Ningen Dock.

[61] Ishizaki T., Wakabayashi M., Tanimoto H., Shima A., Yabune M., Kajimoto O., Itakura H. (2003). Effects of long-term intake of mayonnaise containing phytosterolester on blood cholesterol concentration in Japanese with borderline or mild cholesterolemia. J. Clin. Biochem. Nutr..

[62] Sato H., Chiba Y., Fujimura M., Kondo N., Komai M. (2010). Studies to confirm and serum low-density lipoprotein (LDL) cholesterol level-lowering effect of low-calorie mayonnaise supplemented with plant sterol esters. Jpn. J. Compl. Altern. Med..

[63] Saito S., Takeshita M., Tomonobu K., Kudo N., Shiiba D., Hase T., Tokimitsu I., Yasukawa T. (2006). Dose-dependent cholesterol-lowering effect of a mayonnaise-type product with a main component of diacylglycerol-containing plant sterol esters. Nutrition.

[64] Meguro S., Higashi K., Hase T., Honda Y., Otsuka A., Tokimitsu I., Itakura H. (2001). Solubilization of phytosterols in diacylglycerol versus triacylglycerol improves the serum cholesterol-lowering effect. Eur. J. Clin. Nutr..

[65] Miettinen T.A., Puska P., Gylling H., Vanhanen H., Vartiainen E. (1995). Reduction of serum cholesterol with sitostanol-ester margarine in a mildly hypercholesterolemic population. N. Engl. J. Med..

[66] Homma Y., Ishikawa T., Tateno M., Mitaniyama A., Sugano M. (2000). Cholesterol- and apolipoprotein-lowing effect of plant stanol ester in healthy Japanese men and women. J. Jpn. Soc. Nutr. Food Sci..

[67] Goto N., Mori H., Katsuragi Y., Toi T., Yasukawa T., Shimasaki H. (1999). Effects of diacylglycerol containing phytosterol on reducing blood cholesterol level. J. Jpn. Oil Chem. Soc..

[68] Ho X.L., Liu J.J., Loke W.M. (2016). Plant sterol-enriched soy milk consumption modulates 5-lipoxygenase, 12-lipoxygenase, and myeloperoxidase activities in healthy adults—A randomized-controlled trial. Free Radic. Res..

[69] Chau Y.P., Cheng Y.C., Sing C.W., Tsoi M.F., Cheng V.K., Lee G.K., Cheung C.L., Cheung B.M.Y. (2020). The lipid-lowering effect of once-daily soya drink fortified with phytosterols in normocholesterolaemic Chinese: A double-blind randomized controlled trial. Eur. J. Nutr..

[70] Cheung C.L., Ho D.K., Sing C.W., Tsoi M.F., Cheng V.K., Lee G.K., Ho Y.N., Cheung B.M. (2017). Randomized controlled trial of the effect of phytosterols-enriched low-fat milk on lipid profile in Chinese. Sci. Rep..

[71] Blanco-Morales V., López-García G., Cilla A., Garcia-Llatas G., Barberá R., Lagarda M.J., Sánchez-Siles L.M., Alegría A. (2018). The impact of galactooligosaccharides on the bioaccessibility of sterols in a plant sterol-enriched beverage: Adaptation of the harmonized INFOGEST digestion method. Food Funct..

[72] Devaraj S., Jialal I., Vega-López S. (2004). Plant sterol-fortified orange juice effectively lowers cholesterol levels in mildly hypercholesterolemic healthy individuals. Arterioscler. Thromb. Vasc. Biol..

[73] Li N.Y., Li K., Qi Z., Demonty I., Gordon M., Francis L., Molhuizen H.O., Neal B.C. (2007). Plant sterol-enriched milk tea decreases blood cholesterol concentrations in Chinese adults: A randomized controlled trial. Br. J. Nutr..

[74] Kurokawa M., Masuda Y., Noda M., Marushima R., Usuda M., Takeda S., Hasegawa M., Homma Y., Sugano M. (2008). Minimal effective dose on serum cholesterol concentration and the safety evaluation of dressing containing plant sterol in Japanese subjects. J. Oleo Sci..

[75] Kurokawa M., Masuda Y., Noda M., Usuda M., Takeda S., Hasegawa M., Homma Y., Sugano M. (2008). Effects of dressing containing plant sterol on serum cholesterol concentration and the safety evaluation in borderline or mildly hypercholesterolemic Japanese subjects. J. Oleo Sci..

[76] Elisa T., Miriana S., Davide P., Michele T., Ersilia L., Lara T., Angelo B. (2022). Efficacy of Plant Sterol-Enriched Food for Primary Prevention and Treatment of Hypercholesterolemia: A Systematic Literature Review. Foods.

[77] Zhang R., Han Y., McClements D.J., Xu D., Chen S. (2022). Production, Characterization, Delivery, and Cholesterol-Lowering Mechanism of Phytosterols: A Review. J. Agric. Food. Chem..

[78] Makhmudova U., Schulze P.C., Lütjohann D., Weingärtner O. (2021). Phytosterols and Cardiovascular Disease. Curr. Atheroscler. Rep..

[79] Poli A., Marangoni F., Corsini A., Manzato E., Marrocco W., Martini D., Medea G., Visioli F. (2021). Phytosterols, Cholesterol Control, and Cardiovascular Disease. Nutrient.

[80] Kaur R., Myrie S.B. (2020). Association of Dietary Phytosterols with Cardiovascular Disease Biomarkers in Humans. Lipids.

[81] Köhler J., Teupser D., Elsässer A., Weingärtner O. (2017). Plant sterol enriched functional food and atherosclerosis. Br. J. Pharmacol..

[82] Fumeron F., Bard J.M., Lecerf J.M. (2017). Interindividual variability in the cholesterol-lowering effect of supplementation with plant sterols or stanols. Nutr. Rev..

[83] Prasad M., Jayaraman S., Eladl M.A., El-Sherbiny M., Abdelrahman M.A.E., Veeraraghavan V.P., Vengadassalapathy S., Umapathy V.R., Jaffer Hussain S.F., Krishnamoorthy K. (2022). A Comprehensive Review on Therapeutic Perspectives of Phytosterols in Insulin Resistance: A Mechanistic Approach. Molecules.

[84] Jayaraman S., Roy A., Vengadassalapathy S., Sekar R., Veeraraghavan V.P., Rajagopal P., Rengasamy G., Mukherjee R., Sekar D., Manjunathan R. (2021). An Overview on the Therapeutic Function of Foods Enriched with Plant Sterols in Diabetes Management. Antioxidants.

[85] Vilahur G., Ben-Aicha S., Diaz-Riera E., Badimon L., Padró T. (2019). Phytosterols and Inflammation. Curr. Med. Chem..

